# Fecal microbiota transplantation for patients with ulcerative colitis: a systematic review and meta-analysis of randomized control trials

**DOI:** 10.1007/s10151-025-03113-7

**Published:** 2025-04-17

**Authors:** R. Gefen, J. Dourado, S. H. Emile, A. Wignakumar, P. Rogers, P. Aeschbacher, Z. Garoufalia, N. Horesh, S. D. Wexner

**Affiliations:** 1 Ellen Leifer Shulman and Steven Shulman Digestive Disease Center, Cleveland Clinic Florida, Cleveland Clinic Florida, 2950 Cleveland Clinic Blvd., Weston, FL 33331 USA; 2 Department of General Surgery Hadassah Medical Organization and Faculty of Medicine, Hebrew University of Jerusalem, Jerusalem, Israel; 3 Colorectal Surgery Unit, Mansoura University Hospital, Mansoura University, Mansoura, Egypt; 4 Department of Visceral Surgery and Medicine, Inselspital, Bern University Hospital, University of Bern, Bern, Switzerland; 5 Department of Surgery and Transplantations, Sheba Medical Center, Ramat Gan, Israel

**Keywords:** Fecal microbiota transplantation, Ulcerative colitis, Meta-analysis, Randomized control trials

## Abstract

**Background:**

Fecal microbiota transplantation (FMT) has been shown to restore gut microbiome composition with an acceptable safety profile. FMT in inflammatory bowel disease, specifically ulcerative colitis (UC), has been investigated. We aimed to assess the efficacy of FMT in inducing UC remission.

**Methods:**

PubMed, Scopus, Google Scholar, and clinicaltrials.gov were searched for randomized control trials that assessed FMT in inducing UC remission. The primary outcome was combined clinical and endoscopic remission. Secondary outcomes were clinical remission, endoscopic remission, post-treatment overall adverse events, and colitis. Sensitivity analyses, meta-regression, bias assessment, and grading of certainty of evidence were performed.

**Results:**

A total of 14 studies including 600 patients (55.8% male; median age 40.7 years) were assessed. FMT was used in 299 patients and associated with significantly higher odds of combined clinical and endoscopic remission (OR 2.25, 95% CI 1.54, 3.3; *p* < 0.0001), clinical remission (OR 2.02, 95% CI 1.4, 2.93; *p* = 0.0002), and endoscopic remission (OR 1.95, 95% CI 1.17, 3.28; *p* = 0.011). The odds of post-treatment overall adverse events (OR 1.24, 95% CI 0.79, 1.95; *p* = 0.34) and colitis (OR 0.85, 95% CI 0.52, 1.93; *p* = 0.512) were similar between groups. Compared with baseline, FMT was more effective when biologics (OR 2.71), steroids (OR 2.27), or methotrexate (OR 3.07) were used as pre-FMT treatment. Oral delivery of FMT (OR 3.15) and pooled donors (OR 3.32) led to higher odds of remission. On meta-regression, pooled donors and methotrexate pre-treatment were associated with an increased likelihood of remission.

**Conclusions:**

FMT is promising in inducing UC remission. Administration of medical treatments before FMT may help achieve higher remission rates. Current evidence shows that oral delivery of FMT and multidonor FMT may confer better results.

**Supplementary Information:**

The online version contains supplementary material available at 10.1007/s10151-025-03113-7.

## Introduction[Update Editor corrections from '10151_2025_3113_Rodrigo Fecal Microbiota Tx UC']

Gut microbiota have an important role in the immune system’s development and function. Previous studies have documented a significant alteration of the microbiota composition in patients with inflammatory bowel disease (IBD) [1, 2]. A decrease in butyrate-producing bacteria with an antiinflammatory effect has been shown in patients with IBD [2, 3]. The microbiota composition in patients with IBD may differ during active disease, relapse, and remission phases, which supports that alteration of gut microbiota may contribute to IBD pathogenesis. [1]

The application of fecal microbiota transplantation (FMT) is presumed to rapidly restore the normal composition of the intestinal microbiota. The suggested mechanisms of FMT include altering microbiota dysbiosis, reducing the intestinal permeability, and increasing the production of short-chain fatty acids, which are essential for intestinal wall function [2]. The role of FMT in treating recurrent clostridioides difficile infection (CDI) is well established [2, 4]. Considering its positive results in patients with recurrence CDI, its relative safety, and the evolving evidence linking gut microbiota to many other medical conditions, the use of FMT in treating other gastrointestinal and non-gastrointestinal medical conditions is currently being investigated [4].

Although FMT has been a therapeutic strategy for IBD, especially ulcerative colitis (UC), its efficacy remains unclear. Certain factors may affect the efficacy of FMT, such as the donor criteria, admission route, number of FMT sessions needed, and concurrent use of steroids, biologic, or other antiinflammatory agents. In this meta-analysis we assessed randomized control trials (RCTs) that compared FMT with placebo or standard treatment for patients with UC. The hypothesis of the study is that FMT may confer better remission of UC with comparable side effects to standard medical therapy, and that improvement in symptoms may vary on the basis of patient- and treatment-related factors. The primary aim of this meta-analysis was to assess the efficacy of FMT in patients with UC, stratified by confounding factors such as disease duration, preoperative treatments, sources of FMT, and mode of delivery of FMT.

## Methods

### Registration and reporting

The protocol of this systematic review was prospectively registered in the International Prospective Register of Systematic Reviews (PROSPERO) under the registration number CRD42023478988. The systematic review was reported consistent with the registered protocol with no significant deviations. Reporting of the current review followed the screening guidelines established by the Preferred Reporting Items for Systematic Reviews and Meta-Analyses (PRISMA 2020) [5].

### Search strategy

Two authors (R.G. and S.E.) performed an independent systematic search of the literature for randomized clinical trials (RCTs) that assessed remission of UC after the addition of FMT alone. All evaluated studies must have included a control group of patients who did not receive FMT. The recovered articles were cross-checked between the two reviewers and any disagreements about article selection were resolved by mutual agreement and consensus between the authors. A third author (J.D.) reviewed the agreed-upon list of articles, and a final list of eligible articles was created.

Electronic databases including PubMed and Scopus were searched from their inception through November 2023 without any language restrictions. A parallel search of Google Scholar and the clinical trials registry was conducted. Studies other than randomized clinical trials were excluded. The databases were searched using Medical Subject Headings (MeSH) or the equivalent, title/author key words, truncation, and Boolean operators. Strategies included the terms (fecal microbiota OR FMT OR fecal transplant OR bacteriotherapy) AND (ulcerative colitis OR UC) AND (outcome OR efficacy OR remission OR safety OR complications).

### Assessment of bias

Three authors (P.R., P.A., A.K.) assessed the risk of bias in the studies independently using the risk of bias-2 tool (ROB 2) [6]. Any conflicts of the assessments were reviewed and resolved by another author (J.D.). The certainty of evidence was graded with the GRADE approach as very low, low, moderate, or high [7]. The publication bias in the main outcomes was assessed using the funnel plot method where symmetry of the funnel indicated absence of significant publication bias.

### Data extraction and study outcomes

Two investigators (J.D. and R.G.) extracted the following information from each study:

1. Author, title, journal of publication, publication year, study design, and country of study
2. Number of patients in each group, age, body mass index (BMI), diagnosis, and sex
3. Disease duration in each arm
4. Allowance for medical therapy, including biologics, steroids, 5-aminosalicylic acid (5-ASA), and methotrexate, before FMT and the details of the primary treatment
5. The primary outcome related to efficacy was the combined clinical and endoscopic remission
6. Secondary outcomes related to efficacy were endoscopic remission alone, clinical remission alone, and change in the Mayo score. The secondary outcomes related to safety were post-treatment worsening colitis (clinical diagnosis) and total adverse events.

### Data synthesis

A meta-analysis and meta-regression were conducted using EZR (Easy R) [8] version 1.61 and the open-source, cross-platform software for advanced meta-analysis openMeta [Analyst]^™^ version 12.11.14. A pairwise meta-analysis was conducted to assess the difference in categorical variables including combined remission, clinical remission, and endoscopic remission, and rate of post-treatment colitis expressed as odds ratios (OR) with their respective 95% confidence intervals (CI). Statistical heterogeneity was assessed using the inconsistency (*I*^2^) statistics (low if *I*^2^ < 25%, moderate if *I*^2^ = 25–75%, and high if *I*^2^ > 75%). A common effect meta-analysis was used if the *I*^2^ was low or moderate and *p*-value of heterogeneity > 0.05, and a random-effect analysis was used if *I*^2^ was high. Sensitivity analyses and leave-one-out analysis were performed. *p*-Values < 0.05 were considered significant. Additionally, meta-regression analyses were performed to determine factors significantly (*p* < 0.10) associated with combined clinical and endoscopic remission expressed as the slope coefficient (SE).

## Results

### Description of studies and patients

**Fig. 1:**
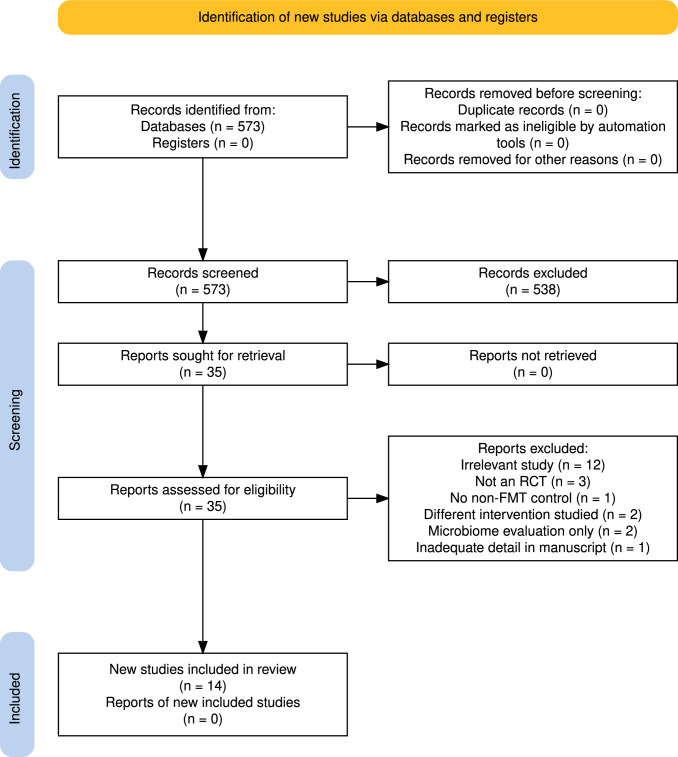
PRISMA Search Flowchart for Study Selection

After screening 573 studies, 14 studies were included in the analysis, all published between 2015 and 2023 (Fig. 1, Table 1); 5 were conducted in Europe, 3 in Australia, another 3 in North America, 2 in Asia, and 1 in Israel. The studies included 600 patients (55.8% male) with a median age of 40.7 (range, 33.8–48) years. Two studies [9, 10] included patients in clinical remission, one [11] allowed patients with Mayo scores ranging from 4-12, two studies [12, 13] simply included active disease, and all other studies included only patients with Mayo scores 3–10. The median duration of UC across all studies was 6 years. The control group was placebo in 10 studies and “standard therapy” in four [11, 14–16]. Delivery of the microbiota was through an oral route in 3 studies [17–19] and transanally in 11 studies. The source of FMT was from pooled donors in seven studies [12, 17–22] and from a single donor in seven studies [9–11, 13–16]. A search of the clinical trial registry revealed 18 active or recruiting trials on FMT for UC (Supplementary Table 1).

**Table 1:** Study characteristics

Study	Location	Year	Study design	No. patients	No. male	Mean age	Control	Delivery of FMT	Donors
Costello et al. [20]	Australia	2019	RCT	73	40	39	Placebo	Endoscopic	Pooled
Sarbagili Shabat et al. [14]	Israel	2021	RCT	62	37	40.4	Diet	Endoscopic	Single
Paramsothy et al. [21]	Australia	2017	RCT	85	47	36.5	Placebo	Endoscopic	Pooled
Pai et al. [12]	Canada	2021	RCT	25			Placebo	Endoscopic	Pooled
Sood et al. [9]	India	2019	RCT	61	44	33.8	Placebo	Endoscopic	Single
Lahtinen et al. [10]	Finland	2023	RCT	33	26	43.1	Placebo	Endoscopic	Single
Fang et al. [11]	China	2021	RCT	20	4	48	Mesalazine/steroid following mesalazine	Endoscopic	Single
Haifer et al. [17]	Australia	2022	RCT	35	18	36.9	Placebo	Oral capsule	Pooled
Karjalainen et al. [13]	Finland	2021	RCT	26	15	44.1	Placebo	Endoscopic + transanal catheter	Single
Schierová et al. [15]	Czechia	2020	RCT	16	8	38.8	5ASA	Enema	Single
Moayyedi et al. [22]	Canada	2015	RCT	75	44	39	Placebo	Enema	Pooled
Crothers et al. [18]	USA	2021	RCT	12	7	46.5	Placebo	Endoscopic + oral capsule	Pooled
Rossen et al. [19]	Amsterdam	2015	RCT	48	22	40.5	Placebo	Nasoduodenal tube	Pooled
Brezina et al. [16]	Czechia	2021	RCT	43	23	42.7	5ASA	Enema	Single

*FMT* fecal microbiota transplantation, *RCT* randomized control trial, *ASA* aminosalicylic acid

FMT was used in 299 patients whereas 301 patients were in the control arms of the studies. The groups were similar in terms of age, sex distribution, and patients with long-standing disease (> 5 years). The “standard therapy” used in the trials was variable and included dietary intervention, mesalazine or steroid following mesalazine, and 5-ASA medications only.

### Efficacy outcomes

**Fig. 2:**
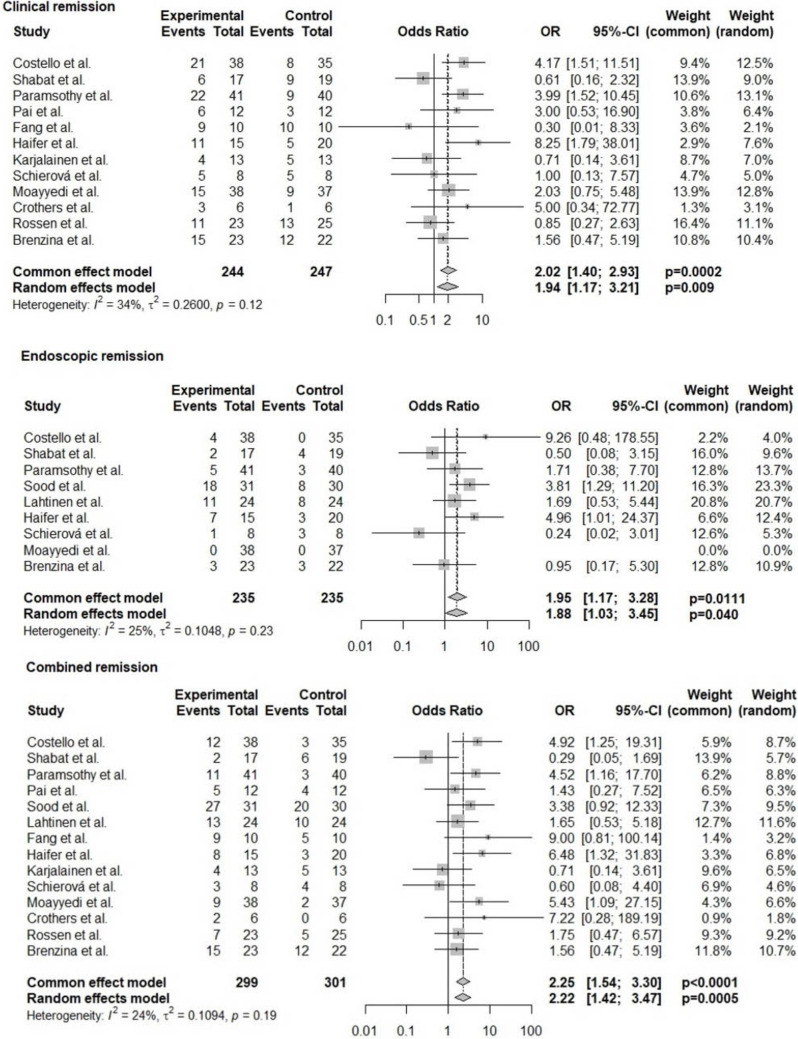
Forest plot depicting the odds of remission (clinical, endoscopic, and combined remission) comparing FMT and control groups

Compared with controls, FMT was associated with significantly higher odds of combined clinical and endoscopic remission (OR 2.25, 95% CI 1.54, 3.3; *p* < 0.0001, *I*^2^ = 24%) (Fig. 2), clinical remission (OR 2.02, 95% CI 1.4, 2.93; *p* = 0.0002, *I*^2^ = 34%) (Fig. 2), and endoscopic remission (OR 1.95, 95% CI 1.17, 3.28; *p* = 0.011, *I*^2^ = 25%) (Fig. 2). The Mayo score was reported by three studies [18, 20, 21], which reported decreased Mayo scores in the FMT group and increased scores in the control group. The median decrease of score in the FMT group was −1.2 (range 0 to −3.4) and the median increase of score in the control group was 0.25 (range 0 to −3.5).

### Safety outcomes

**Fig. 3:**
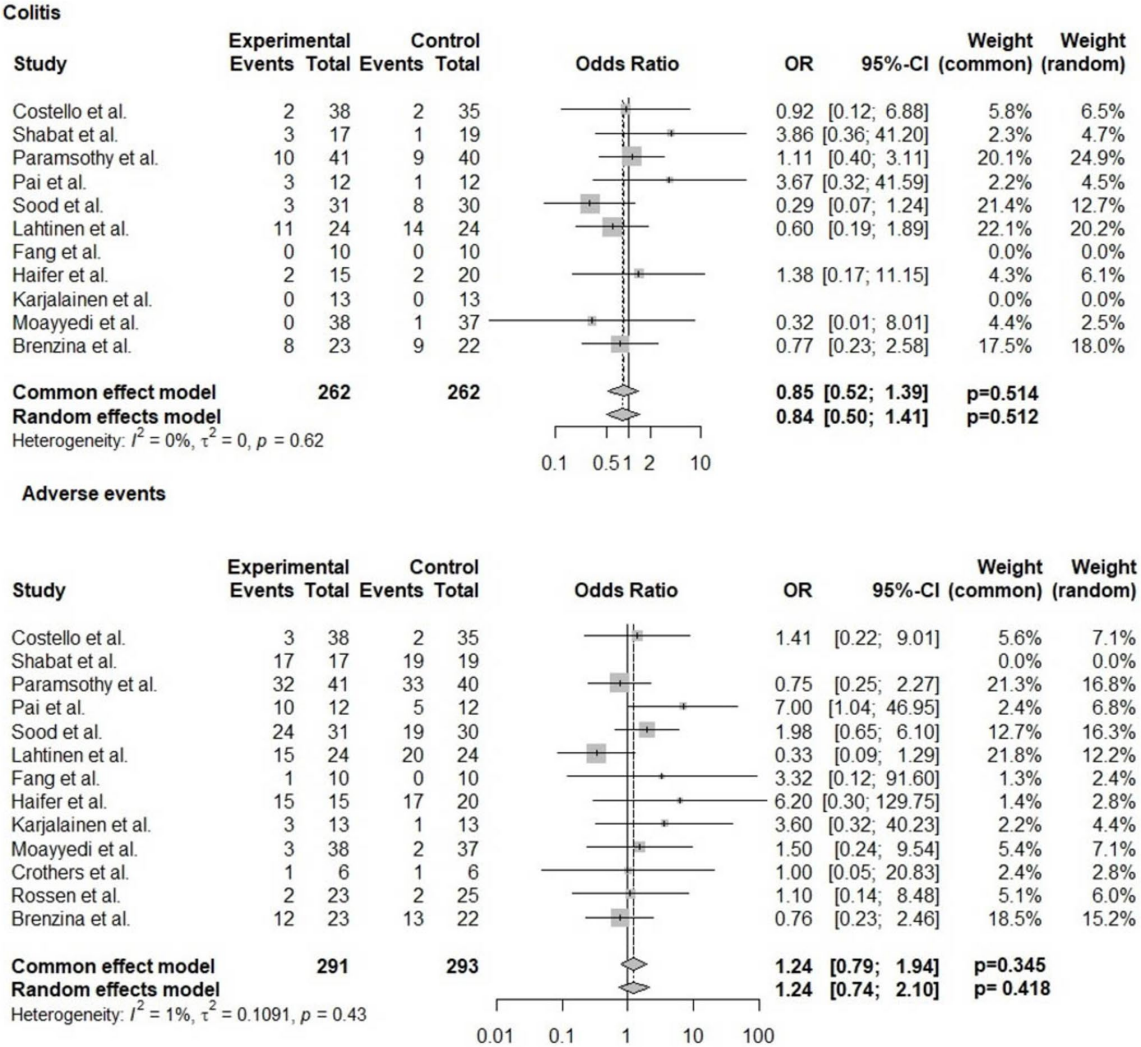
Forest plot depicting the odds of adverse events and colitis comparing FMT and control groups

The most common adverse event was gastrointestinal upset, including bloating, diarrhea, and abdominal pain; however, all these events were resolved conservatively. There were limited reports of serious adverse events. The odds of post-treatment colitis were similar between the FMT and control groups (OR 0.85, 95% CI 0.52, 1.93; *p* = 0.512, *I*^2^ = 0%). Both groups had similar odds of adverse events (OR 1.24, 95% CI 0.79, 1.95; *p* = 0.34, *I*^2^ = 1%) (Fig. 3).

### Sensitivity analysis

#### Control group

When the control group included the use of a placebo agent, FMT was also associated with higher odds of combined clinical and endoscopic remission (OR 2.82, 95% CI 1.80 4.43; *p* < 0.001, *I*^2^ = 0%), clinical remission (OR 2.55, 95% CI 1.44, 4.51; *p* = 0.001, *I*^2^ = 33%), and endoscopic remission (OR 2.94, 95% CI 1.59, 5.43; *p* < 0.001, *I*^2^ = 0%) with no difference in the odds of colitis between the two groups (OR 0.78, 95% CI 0.44, 1.37; *p* = 0.388, *I*^2^ = 0%).

When the control group was composed of “standard therapy,” the odds of combined clinical and endoscopic remission (OR 1.25, 95% CI 0.59, 2.67; *p* = 0.562, *I*^2^ = 52%), clinical remission (OR 0.94, 95% CI 0.43, 2.05; *p* = 0.879, *I*^2^ = 0%), endoscopic remission (OR 0.56, 95% CI 0.19, 1.68; *p* = 0.301, *I*^2^ = 0%), post-treatment colitis (OR 1.12, 95% CI 0.40, 3.17; *p* = 0.824, *I*^2^ = 29%), and adverse events (OR 0.92, 95% CI 0.31, 2.72; *p* = 0.878, *I*^2^ = 0) were similar.

##### Allowance for other treatments

There was a significant variation in which adjunct treatments were allowed concomitant or before the delivery of FMT. All interventions were compared in their ability to achieve combined clinical and endoscopic remission in combination with FMT. The odds of combined remission were significantly increased when biologics were allowed. [12, 14, 17, 18, 20, 22] (OR 2.71, 95% CI 1.00, 7.30) compared with when they were not allowed (OR 1.96, 95% CI 1.18, 3.24), with an estimated 75% increased odds of remission.

The odds of combined remission were significantly increased when steroids were allowed [14–17, 19, 22] [(OR 2.27, 95% CI 1.12, 4.6) compared with when steroid therapy was not allowed (OR 2.03, 95% CI 1.01, 3.88)], with an estimated 24% increased odds of remission.

Similarly, there was an increase in the odds of combined remission when methotrexate was allowed [9, 12, 14, 17, 18, 20, 22] (OR 3.07, 95% CI 1.55, 6.06) compared with when it was not allowed (OR 1.49, 95% CI 0.82, 2.7), with an estimated 158% increased odds of remission. When no pre-FMT treatments were allowed at all [10, 13], the odds of combined remission with FMR were similar to those of controls (OR 1.24, 95% CI 0.49, 3.11, *p* = 0.641, *I*^2^ = 0%).

Two studies [13, 17] allowed pre-treatment antibiotics. The odds of combined remission were lower when pre-treatment antibiotics were administered compared with when they were not given [(OR 2.31, 95% CI 1.52, 3.50) versus (OR 2.4, 95% CI 1.48, 3.88)], with an estimated decreased odds of remission of 9%. These results are summarized in Table 2.

**Table 2:** Sensitivity analyses of the odds of remission with adjunctive treatments in addition to FMT

Treatment	Allowed	Not allowed	Change in odds of remission
Biologics	2.71 (1.00, 7.30)	1.96 (1.18, 3.24)	75%
Steroids	2.27 (1.12, 4.6)	2.03 (1.01, 3.88)	24%
Methotrexate	3.07 (1.55, 6.06)	1.49 (0.82, 2.7)	158%
Antibiotics	2.16 (0.25, 18.81)	2.4 (1.48, 3.88)	− 9%
Any treatment		1.244 (0.489, 3.105)	

*CI* confidence interval, *OR* ratio odds

*OR (95% CI)

##### Duration of disease, mode of delivery, and source of FMT

Patients with chronic disease (> 5 years) [11–14, 16, 17, 19–21] had higher odds of combined remission than in all patients [(OR 2.89, 95% CI 1.51, 5.55) versus OR 2.25, 95% CI 1.54, 3.3]. Oral delivery of FMT [17–19] was associated with increased odds of combined remission compared with transanal delivery [(OR 3.15, 95% CI 1.29, 7.67) versus (OR 2.01, 95% CI 1.16, 3.49)]. The odds of combined remission also increased when the source of FMT was from pooled donors [12, 17–22] compared with when prepared from a single donor [(OR 3.32, 95% CI 1.99, 5.55) versus (OR 1.39, 95% CI 0.69, 2.77)]. The results of the sensitivity analyses are summarized in Table 3.

**Table 3:** Sensitivity analyses for other factors in combination with FMT

Factor	Group	Odds of remission
Disease duration	< 5 years	2.31 (1.41, 3.81)
	> 5 years	2.89 (1.51, 5.55)
Mode of delivery	Transanal	2.01 (1.16, 3.49)
	Oral	3.15 (1.29, 7.67)
Source of FMT	Single	1.39 (0.69, 2.77)
	Pooled	3.32 (1.99, 5.55)

*OR (95% CI)

*FMT* fecal microbiota transplantation, *OR* odds ratio, *CI* confidence interval

##### Leave-one-out analysis

A leave-one-out meta-analysis of combined clinical and endoscopic remission did not show a large effect of any individual study on the odds of achieving remission with FMT. All results remained significant when each study was omitted (Supplementary Fig. 1).

### Meta-regression

A meta-regression analysis of factors associated with combined clinical and endoscopic remission showed the use of pooled donors (SE 0.921; *p* = 0.026) and pre-treatment with methotrexate (SE 0.744; *p* = 0.073) were significantly associated with an increased likelihood of remission (Table 4).

**Table 4:** Meta regression for factors associated with combined clinical and endoscopic remission

Factor	SE	*p*-Value
Age	−0.053	0.345
Male sex	0.059	0.109
Oral versus transanal delivery	0.460	0.348
Single versus pooled donor	0.921	**0.026**
Disease duration	−0.149	0.111
Pre-treatment steroids	0.146	0.733
Pre-treatment biologics	0.358	0.411
Pre-treatment methotrexate	0.744	**0.073**
Pre-treatment antibiotics	−1.214	0.156

Bold text in *p*-value column indicates statistical significance

*SE* slope coefficient

### Risk of bias and certainty of evidence

The risk of bias was low for five studies [13–15, 17, 20], whereas nine studies had some concerns of bias (Supplementary Fig. 2) [9–12, 16, 18, 19, 21, 22]. The certainty of evidence was high for combined remission and clinical remission and moderate for endoscopic remission, overall adverse events and colitis (Supplementary Fig. 3). There was no significant publication bias in the primary outcomes (Supplementary Fig. 4).

## Discussion

In this meta-analysis, 299 patients with UC were treated with FMT. FMT was associated with higher rates of clinical and endoscopic remission. Patients treated with steroids, biological agents, or methotrexate before FMT had higher likelihood of remission compared with patients who did not receive such treatments. Interestingly, in the studies that did not allow pre-FMT treatments there was no significant advantage of FMT, which may imply beneficial effect of pre-FMT treatments in preparing the bowel for the bacteriotherapy. Oral delivery of FMT prepared from multiple donors conferred higher remission than transanal delivery and single-donor FMT.

Various methods of preparing and administering FMT have been described. Postigo and Kim [23] demonstrated that delivery of FMT via a nasogastric tube was equally effective to colonoscopy in recurrent CDI. Similarly, in an RCT Kao et al. found that oral capsules were not inferior to colonoscopy as a method of FMT delivery [24]. These prior results were consistent with our finding that oral administration FMT yielded higher remission of UC. This finding is clinically important because oral delivery of FMT is easier and less invasive than transanal delivery. In addition, oral delivery may facilitate a daily administration, allowing for better compliance with therapy and potentially maintenance therapy [17]. Therefore, future trials may opt to focus more on oral FMT, as it combines both efficacy and compliance.

Further consideration of FMT is the source of microbiota and whether it is better to use a single-donor or a multidonor sample. Our results support the multidonor approach, as it led to a higher remission than the single-donor approach. A possible explanation is that multidonor FMT has a larger microbial diversity, as shown by Paramsothy and colleagues [21]. In addition, Vermeire et al. [25] demonstrated that higher sample richness is important in achieving remission.

An important finding of the present meta-analysis is that the likelihood of remission after FMT was increased in patients who received biological treatment, steroids, or methotrexate before or concomitant with FMT. Recent studies have cast doubt on methotrexate’s efficacy in inducing and maintaining remission [26–28]. Despite these studies, its use is still recommended by the American Gastroenterological Association (AGA) combined with biological agents rather than biological monotherapy [28]. The role of methotrexate in combination with FMT requires further evaluation. Aden et al. [29] concluded that anti-TNF treatment induces the restoration of intestinal microbial diversity in patients with IBD. The authors assumed that biological therapy might affect the intra-microbiota interaction, thus improving the efficacy of FMT.

Fukushima et al. [3] investigated the relationship between the use of biological antiinflammatory drugs (anti TNF agents) and the gut microbiota of patients with Crohn’s disease (CD). Their study found that the gut microbiota diversity was significantly different in patients with CD compared with control patients, however, there was no significant difference between those who received anti-TNF therapy and those who did not.

As has emerged from the literature, it is unclear whether biological and other antiinflammatory agents influence intra-microbiota interaction and thus increase the efficacy of FMT treatment. A different explanation suggests it is simply an example of the multifactorial pathogenesis of IBD, in which case it is beneficial to target concurrently different mechanisms—antiinflammatory drugs and FMT—to increase remission rates.

Pre-treatment antibiotics are theoretically supposed to clean the gastrointestinal tract and facilitate the engraftment of donor microbes. Mocanu et al. [30] showed in their proportional meta-analysis that antibiotic treatment prior to FMT improved remission rates in patients with IBD. Conversely, we found that giving antibiotics prior to FMT decreased the odds of remission. However, the small number of patients included in this analysis (only two papers included) may prevent drawing definitive conclusions.

Previous studies on recurrent CDI [23] and IBD [4, 30] have shown that FMT is relatively safe. Consistent with former studies, we found that the odds for post-treatment colitis were similar between FMT and control groups, and there was no compromise in safety profile when FMT was used.

This meta-analysis included only randomized control trials to ensure a high level of evidence and minimize selection bias. In addition, this analysis included a relatively large number of patients. However, the study has several limitations. It is important to remember that the study design, FMT preparation, administration, and FMT regimens varied between the studies. While this heterogeneity was indeed a major limitation when conducting the primary analysis of all studies, it helped us stratify studies and patients according to the delivery method, source of FMT, and pre-FMT treatment. Eventually, the results of these sensitivity analyses can guide future studies and inform clinical practice, such as oral delivery of FMT, multidonor FMT, and administration of medical treatments before FMT that may be recommended to achieve higher remission of UC. However, there were no discreet data on which patients received which pre-treatments, only that pre-treatment was allowed in certain studies. This limits the ability to concretely associate a specific pre-treatment with increased remission. Moreover, the assertion that antibiotics diminish remission probabilities is not conclusive owing to the limited sample size of this analysis. Another limitation related to the different regimens is that, although most of the regimens included more than a single FMT administration, we were unable to determine the optimal number of FMT delivery. Furthermore, the safety profile of FMT was not a major focus of the included studies.

## Conclusions

FMT has a promising role in inducing remission of UC. The administration of medical treatments before FMT may help achieve higher remission of UC and thus FMT may be associated with better results if not used as a single treatment method. Current evidence shows that oral delivery of FMT and multidonor FMT may confer better results than transanal delivery and single-donor FMT. Standardization of the administration method, dosage, donors, and pre-FMT treatment is needed.

## Supplementary Information

Below is the link to the electronic supplementary material.

Supplementary file1 Supplementary Fig. 1: Leave-one-out analysis (JPG 70 KB)

Supplementary file2 Supplementary Fig. 2: Assessment of risk of bias using the RoB 2 tool (JPG 189 KB)

Supplementary file3 Supplementary Fig. 3: GRADE analysis of certainty of evidence (JPG 141 KB)

Supplementary file4 Supplementary Fig. 4: Assessment of publication bias for all major outcomes (JPG 81 KB)

Supplementary file5 (DOCX 16 KB)

## Data Availability

Data available upon reasonable request from co-first authors: rachel.gefen@mail.huji.ac.il or douradoj@health.fau.edu.

## References

[1] Nishida A, Inoue R, Inatomi O et al. (2018) Gut microbiota in the pathogenesis of inflammatory bowel disease. Clin J Gastroenterol 11:1–10. 10.1007/s12328-017-0813-5 29285689 10.1007/s12328-017-0813-5

[2] Shen ZH, Zhu CX, Quan YS et al. (2018) Relationship between intestinal microbiota and ulcerative colitis: mechanisms and clinical application of probiotics and fecal microbiota transplantation. World J Gastroenterol 24:5–14. 10.3748/wjg.v24.i1.5.PMID:29358877;PMCID:PMC5757125 29358877 10.3748/wjg.v24.i1.5 PMC5757125

[3] Fukushima S, Shiotani A, Matsumoto H et al. (2022) Comparison of mucosa-associated microbiota in Crohn’s disease patients with and without anti-tumor necrosis factor-α therapy. J Clin Biochem Nutr 270:182–188. 10.3164/jcbn.21-41 10.3164/jcbn.21-41 PMC8921723 35400819

[4] Ooijevaar RE, Terveer EM, Verspaget HW, Kuijper EJ, Keller JJ (2019) Clinical application and potential of fecal microbiota transplantation. Annu Rev Med 70:335–351. 10.1146/annurev-med-111717-122956. (**Epub 2018 Nov 7 PMID: 30403550**) 30403550 10.1146/annurev-med-111717-122956

[5] Page MJ, McKenzie JE, Bossuyt PM et al. (2021) The PRISMA 2020 statement: an updated guideline for reporting systematic reviews. BMJ 372:n71 33782057 10.1136/bmj.n71 PMC8005924

[6] Sterne JAC, Savović J, Page MJ et al. (2019) RoB 2: a revised tool for assessing risk of bias in randomised trials. BMJ 19(366):l4898 10.1136/bmj.l4898 31462531

[7] Balshem H, Helfand M, Schunemann HJ et al. (2011) GRADE guidelines: 3. Rating the quality of evidence. J Clin Epidemiol 64:401–406 21208779 10.1016/j.jclinepi.2010.07.015

[8] Kanda Y (2013) Investigation of the freely available easy-to-use software “EZR” for medical statistics. Bone Marrow Transplant 48:452–458. 10.1038/bmt.2012.244 23208313 10.1038/bmt.2012.244 PMC3590441

[9] Sood A, Mahajan R, Singh A et al. (2019) Role of faecal microbiota transplantation for maintenance of remission in patients with ulcerative colitis: a pilot study. J Crohns Colitis 13:1311–1317. 10.1093/ecco-jcc/jjz060 30873549 10.1093/ecco-jcc/jjz060

[10] Lahtinen P, Jalanka J, Mattila E et al. (2023) Fecal microbiota transplantation for the maintenance of remission in patients with ulcerative colitis: a randomized controlled trial. World J Gastroenterol 29:2666–2678. 10.3748/wjg.v29.i17.2666 37213403 10.3748/wjg.v29.i17.2666 PMC10198050

[11] Fang H, Fu L, Li X et al. (2021) Long-term efficacy and safety of monotherapy with a single fresh fecal microbiota transplant for recurrent active ulcerative colitis: a prospective randomized pilot study. Microb Cell Fact 20:18. 10.1186/s12934-021-01513-6 33468164 10.1186/s12934-021-01513-6 PMC7816432

[12] Pai N, Popov J, Hill L et al. (2021) McMaster pediatric fecal microbiota transplant research collaboration. results of the first pilot randomized controlled trial of fecal microbiota transplant in pediatric ulcerative colitis: lessons, limitations, and future prospects. Gastroenterology 161:388-393.e3. 10.1053/j.gastro.2021.04.067 33961887 10.1053/j.gastro.2021.04.067

[13] Karjalainen EK, Renkonen-Sinisalo L, Satokari R et al. (2021) Fecal microbiota transplantation in chronic pouchitis: a randomized, parallel, double-blinded clinical trial. Inflamm Bowel Dis 27:1766–1772. 10.1093/ibd/izab001 33501942 10.1093/ibd/izab001 PMC8528148

[14] Sarbagili Shabat C, Scaldaferri F, Zittan E et al. (2022) Use of faecal transplantation with a novel diet for mild to moderate active ulcerative colitis: the CRAFT UC randomised controlled trial. J Crohns Colitis 16:369–378. 10.1093/ecco-jcc/jjab165 34514495 10.1093/ecco-jcc/jjab165 PMC8919830

[15] Schierová D, Březina J, Mrázek J, Fliegerová KO, Kvasnová S, Bajer L, Drastich P (2020) Gut microbiome changes in patients with active left-sided ulcerative colitis after fecal microbiome transplantation and topical 5-aminosalicylic acid therapy. Cells 9:2283. 10.3390/cells9102283 33066233 10.3390/cells9102283 PMC7602113

[16] Březina J, Bajer L, Wohl P et al. (2021) Fecal microbial transplantation versus mesalamine enema for treatment of active left-sided ulcerative colitis-results of a randomized controlled trial. J Clin Med 10:2753. 10.3390/jcm10132753.PMID:34206663;PMCID:PMC8268406 34206663 10.3390/jcm10132753 PMC8268406

[17] Haifer C, Paramsothy S, Kaakoush NO et al. (2022) Lyophilised oral faecal microbiota transplantation for ulcerative colitis (LOTUS): a randomised, double-blind, placebo-controlled trial. Lancet Gastroenterol Hepatol 7:141–151. 10.1016/S2468-1253(21)00400-3. (**Epub 2021 Dec 2 PMID: 34863330**) 34863330 10.1016/S2468-1253(21)00400-3

[18] Crothers JW, Chu ND, Nguyen LTT et al. (2021) Daily, oral FMT for long-term maintenance therapy in ulcerative colitis: results of a single-center, prospective, randomized pilot study. BMC Gastroenterol 21:281. 10.1186/s12876-021-01856-9 34238227 10.1186/s12876-021-01856-9 PMC8268596

[19] Rossen NG, Fuentes S, van der Spek MJ et al. (2015) Findings from a randomized controlled trial of fecal transplantation for patients with ulcerative colitis. Gastroenterology 149:110-118.e4. 10.1053/j.gastro.2015.03.045 25836986 10.1053/j.gastro.2015.03.045

[20] Costello SP, Hughes PA, Waters O et al. (2019) Effect of fecal microbiota transplantation on 8-week remission in patients with ulcerative colitis: a randomized clinical trial. JAMA 321:156–164. 10.1001/jama.2018.20046 30644982 10.1001/jama.2018.20046 PMC6439766

[21] Paramsothy S, Kamm MA, Kaakoush NO et al. (2017) Multidonor intensive faecal microbiota transplantation for active ulcerative colitis: a randomised placebo-controlled trial. Lancet 389:1218–1228. 10.1016/S0140-6736(17)30182-4. (**Epub 2017 Feb 15 PMID: 28214091**) 28214091 10.1016/S0140-6736(17)30182-4

[22] Moayyedi P, Surette MG, Kim PT et al. (2015) Fecal microbiota transplantation induces remission in patients with active ulcerative colitis in a randomized controlled trial. Gastroenterology 149:102-109.e6. 10.1053/j.gastro.2015.04.001. (**Epub 2015 Apr 7 PMID: 25857665**) 25857665 10.1053/j.gastro.2015.04.001

[23] Postigo R, Kim JH (2012) Colonoscopic versus nasogastric fecal transplantation for the treatment of Clostridium difficile infection: a review and pooled analysis. Infection 40:643–648. 10.1007/s15010-012-0307-9. (**Epub 2012 Jul 31 PMID: 22847629**) 22847629 10.1007/s15010-012-0307-9

[24] Kao D, Roach B, Silva M et al. (2017) Effect of oral capsule- vs colonoscopy-delivered fecal microbiota transplantation on recurrent clostridium difficile infection: a randomized clinical trial. JAMA 318:1985–1993. 10.1001/jama.2017.17077.PMID:29183074;PMCID:PMC5820695 29183074 10.1001/jama.2017.17077 PMC5820695

[25] Vermeire S, Joossens M, Verbeke K et al. (2016) Donor species richness determines faecal microbiota transplantation success in inflammatory bowel disease. J Crohns Colitis 10:387–394. 10.1093/ecco-jcc/jjv203 26519463 10.1093/ecco-jcc/jjv203 PMC4946755

[26] Herfarth H, Barnes EL, Valentine JF, Hanson J, Higgins PDR, Isaacs KL, Jackson S, Osterman MT, Anton K, Ivanova A, Long MD, Martin C, Sandler RS, Abraham B, Cross RK, Dryden G, Fischer M, Harlan W, Levy C, McCabe R, Polyak S, Saha S, Williams E, Yajnik V, Serrano J, Sands BE, Lewis JD, Clinical Research Alliance of the Crohn’s and Colitis Foundation (2018) Methotrexate is not superior to placebo in maintaining steroid-free response or remission in ulcerative colitis. Gastroenterology 155(4):1098-1108.e9. 10.1053/j.gastro.2018.06.046 29964043 10.1053/j.gastro.2018.06.046 PMC6174092

[27] Carbonnel F, Colombel JF, Filippi J, Katsanos KH, Peyrin-Biroulet L, Allez M, Nachury M, Novacek G, Danese S, Abitbol V, Bossa F, Moreau J, Bommelaer G, Bourreille A, Fumery M, Roblin X, Reinisch W, Bouhnik Y, Brixi H, Seksik P, Malamut G, Färkkilä M, Coulibaly B, Dewit O, Louis E, Deplanque D, Michetti P, Sarter H, Laharie D, European Crohn’s and Colitis Organisation; Groupe d’Étude Thérapeutique des Affections Inflammatoires Digestives (2016) Methotrexate is not superior to placebo for inducing steroid-free remission, but induces steroid-free clinical remission in a larger proportion of patients with ulcerative colitis. Gastroenterology 150(2):380–8.e4. 10.1053/j.gastro.2015.10.050 26632520 10.1053/j.gastro.2015.10.050

[28] Feuerstein JD, Isaacs KL, Schneider Y, Siddique SM, Falck-Ytter Y, Singh S, AGA Institute Clinical Guidelines Committee (2020) AGA clinical practice guidelines on the management of moderate to severe ulcerative colitis. Gastroenterology 158(5):1450–1461. 10.1053/j.gastro.2020.01.006 31945371 10.1053/j.gastro.2020.01.006 PMC7175923

[29] Aden K, Rehman A, Waschina S et al. (2019) Metabolic functions of gut microbes associate with efficacy of tumor necrosis factor antagonists in patients with inflammatory bowel diseases. Gastroenterology 157:1279–1292. 10.1053/j.gastro.2019.07.025. (**Epub 2019 Jul 18 PMID: 31326413**) 31326413 10.1053/j.gastro.2019.07.025

[30] Mocanu V, Rajaruban S, Dang J, Kung JY, Deehan EC, Madsen KL (2021) Repeated fecal microbial transplantations and antibiotic pre-treatment are linked to improved clinical response and remission in inflammatory bowel disease: a systematic review and pooled proportion meta-analysis. J Clin Med 10:959. 10.3390/jcm10050959.PMID:33804464;PMCID:PMC7957789 33804464 10.3390/jcm10050959 PMC7957789

