# Supplementary figures and images for: Fecal microbiota transplantation for patients with ulcerative colitis: a systematic review and meta-analysis of randomized control trials
Source: Tech Coloproctol. 2025 Apr 17;29(1):103. doi: 10.1007/s10151-025-03113-7 (PMC12006273; doi:10.1007/s10151-025-03113-7)

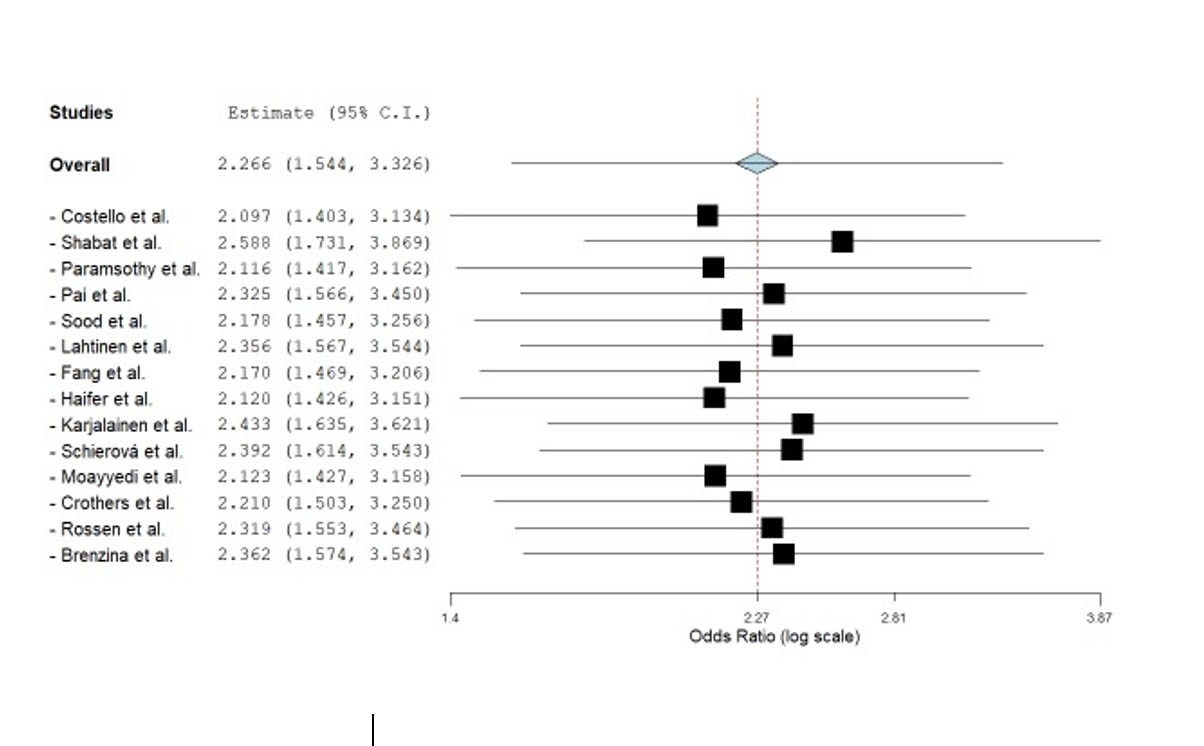

Supplement: Supplementary file 1 — Supplementary file1 Supplementary Fig. 1: Leave-one-out analysis (JPG 70 KB) [file 10151_2025_3113_MOESM1_ESM.jpg]

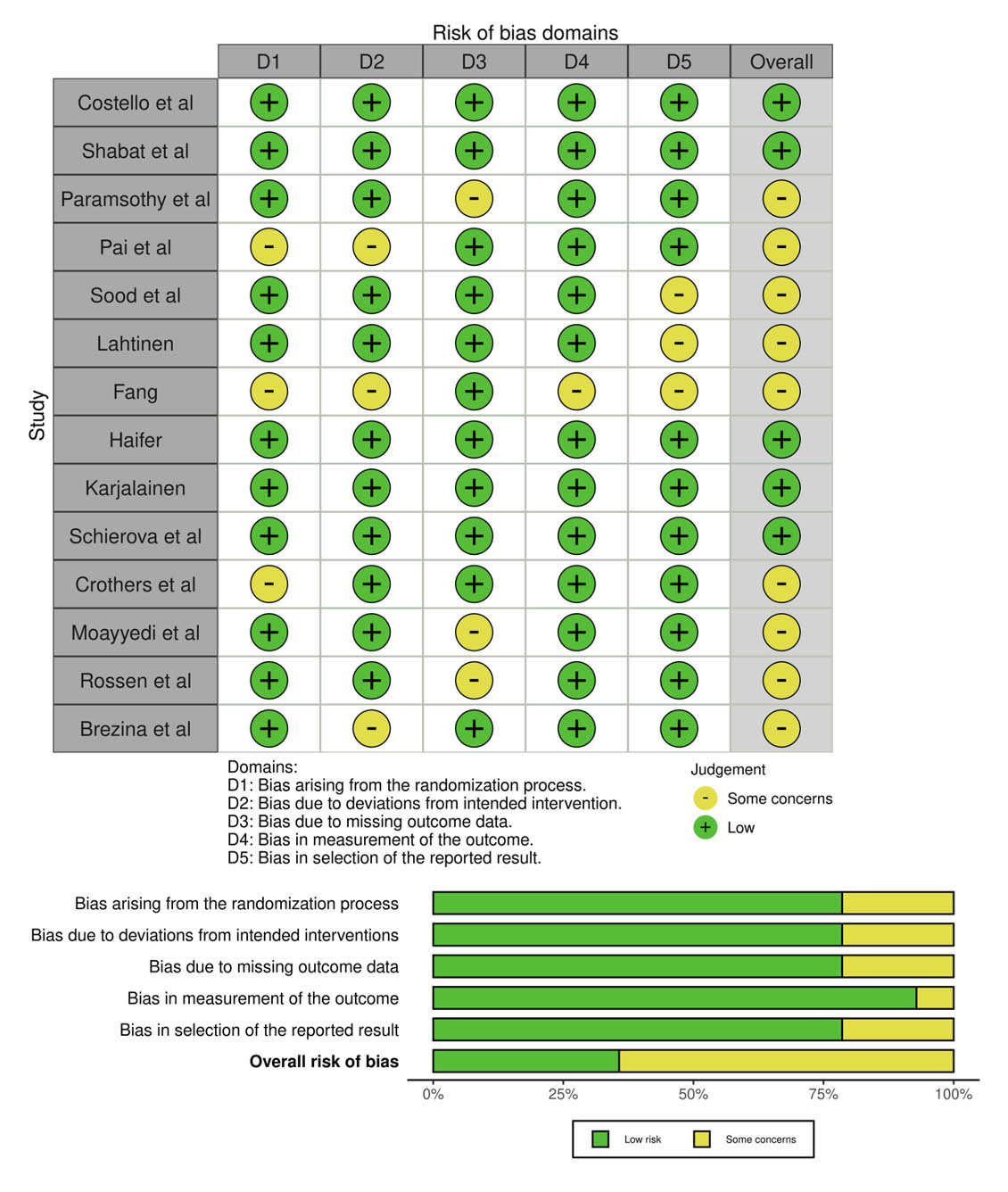

Supplement: Supplementary file 2 — Supplementary file2 Supplementary Fig. 2: Assessment of risk of bias using the RoB 2 tool (JPG 189 KB) [file 10151_2025_3113_MOESM2_ESM.jpg]

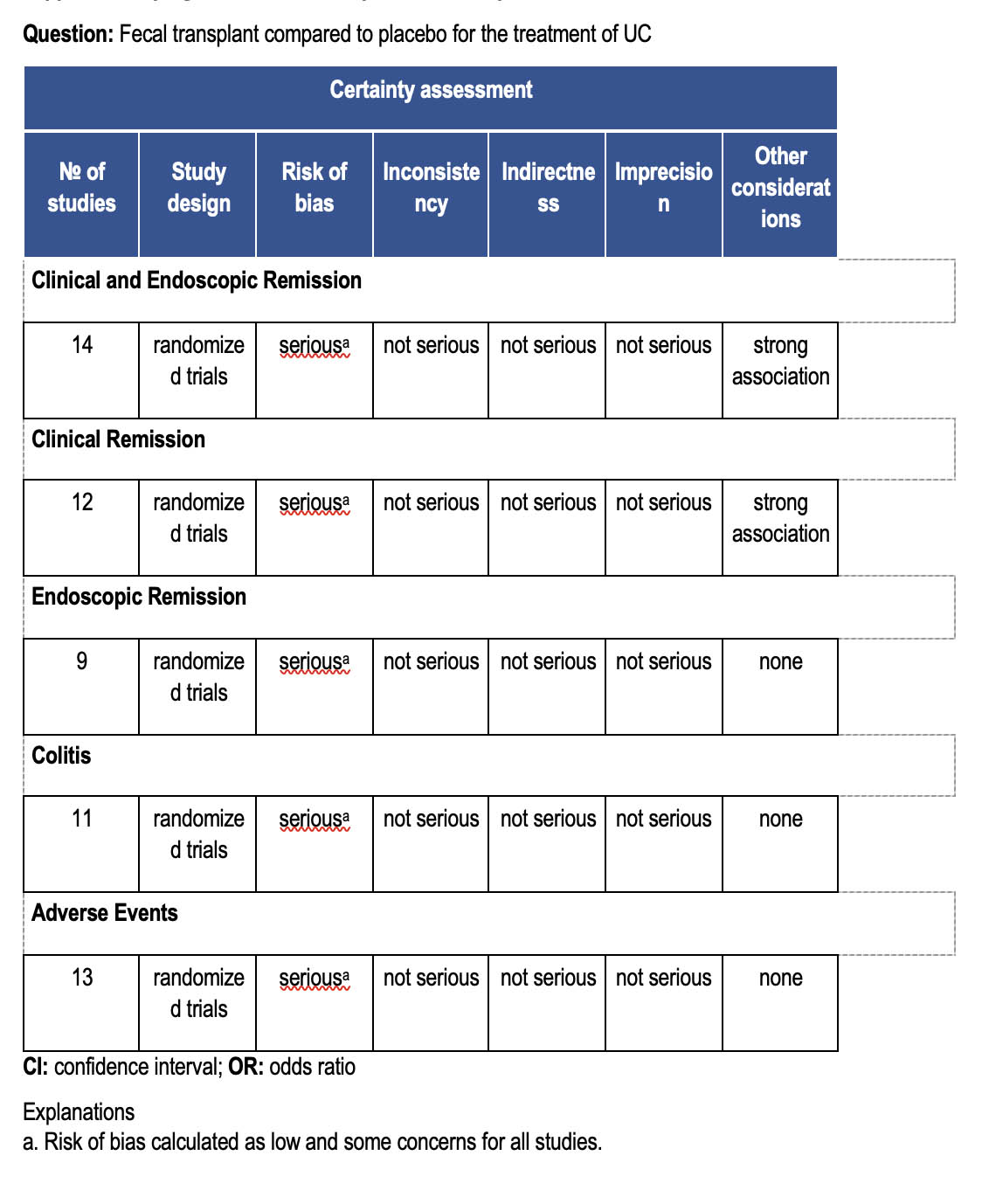

Supplement: Supplementary file 3 — Supplementary file3 Supplementary Fig. 3: GRADE analysis of certainty of evidence (JPG 141 KB) [file 10151_2025_3113_MOESM3_ESM.jpg]

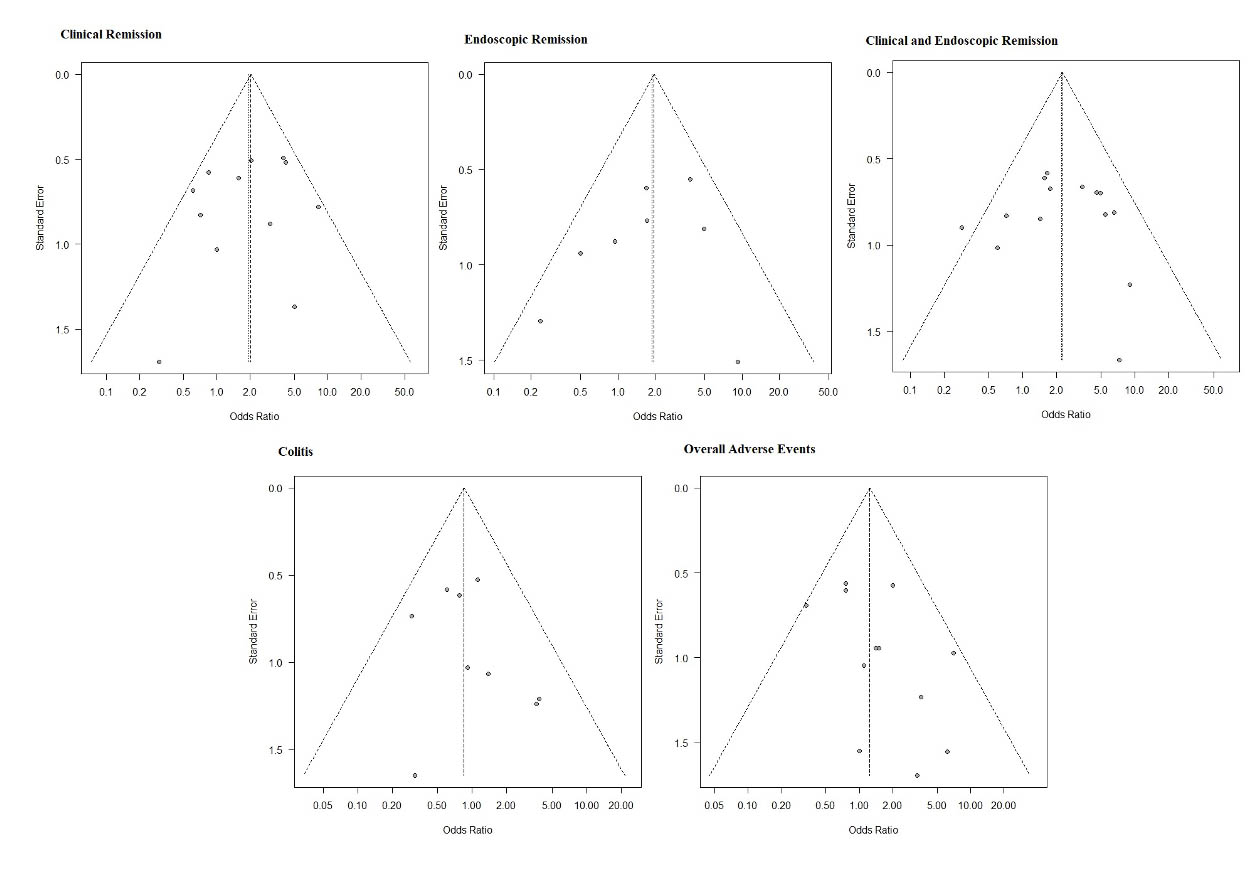

Supplement: Supplementary file 4 — Supplementary file4 Supplementary Fig. 4: Assessment of publication bias for all major outcomes (JPG 81 KB) [file 10151_2025_3113_MOESM4_ESM.jpg]
