# Supplementary material for: Fecal microbiota transplantation for patients with ulcerative colitis: a systematic review and meta-analysis of randomized control trials
Source: Tech Coloproctol. 2025 Apr 17;29(1):103. doi: 10.1007/s10151-025-03113-7 (PMC12006273; doi:10.1007/s10151-025-03113-7)
Supplement: Supplementary file 5 — Supplementary file5 (DOCX 16 KB) [file 10151_2025_3113_MOESM5_ESM.docx]

**Supplementary Table 1: Ongoing Trials of Fecal microbiota transplantation in ulcerative colitis**

| **Title** | **NCT** | **Location** | **Status** |
| --- | --- | --- | --- |
| Transfer of Feces in Ulcerative Colitis 2 | NCT 05998213 | Amsterdam, Netherlands | Recruiting |
| Combination Therapy with Fecal Microbiota Transplantation and Vedolizumab for Induction of Ulcerative Colitis | NCT 04231110 | Ontario, Canada | Recruiting |
| Impact of Fecal Microbiota Transplantation in Ulcerative Colitis | NCT 03483246 | Paris, France | Recruiting |
| Low Sulfur Fecal Transplant for Ulcerative Colitis | NCT 03948919 | Minnesota, USA | Active |
| Fecal Microbiota Transplantation and Newly Diagnosed Ulcerative Colitis | NCT 04687150 | Turku, Finland | Recruiting |
| Fecal Microbiome Transplant | NCT 02636517 | Philadelphia, USA | Recruiting |
| Refined Fecal Microbiota Transplantation for Ulcerative Colitis | NCT 04968951 | New York, USA | Recruiting |
| LFMT vs Placebo in New Biologic Start for Ulcerative Colitis | NCT 05327790 | Alberta, Canada | Recruiting |
| A Multicenter Clinical Trial: Efficacy, Safety of Fecal Microbiota Transplantation for Inflammatory Bowel Disease | NCT 04521205 | Xiamen, China | Recruiting |
| Examining the Efficacy of Fecal Microbiota Transplantation and Dietary Fiber in Patients with Ulcerative Colitis | NCT 03998488 | New York, USA | Active |
| Standardized Fecal Microbiota Transplantation for Ulcerative Colitis | NCT 01790061 | Nanjing, China | Recruiting |
| Fecal Microbiota Transplantation for Ulcerative Colitis | NCT 03804931 | Guangzhou, China | Recruiting |
| The Effect of Therapeutic Fecal Transplant on the Cut Microbiome in Children with Ulcerative Colitis | NCT 02291523 | Los Angeles, USA | Active |
| Safety and Efficacy of Fecal Microbiota Transplantation | NCT 04014413 | Hong Kong, Hong, Kong | Recruiting |
| FMT in Patients with Recurrent CDT and Ulcerative Colitis: Single Infusion Versus Sequential Approach | NCT 06071312 | Rome, Italy | Recruiting |
| Superdonor FMT in Patients with Ulcerative Colitis | NCT 05739864 | Roma, Italy | Recruiting |
| ICON-2: FMT and Bezlotoxumab Compared to FMT and Placebo for Patients with IBD and CDI | NCT 03829475 | Boston, USA | Recruiting |
| Evaluation the Safety and Efficacy of Lyophilized Fecal Microbiota Transplantation administered Orally for Prevention of Relapse of Intestinal Inflammation in Adults with Ulcerative Colitis | NCT 04373473 | Houston, USA | Recruiting |
